# The Combination of α‐Fe_2_O_3_ NP and *Trichoderma* sp. Improves Antifungal Activity Against *Fusarium* Wilt

**DOI:** 10.1002/jobm.202400613

**Published:** 2025-01-19

**Authors:** Sushma Sharma, Poonam Kumari, Mamta Shandilya, Sapna Thakur, Kahkashan Perveen, Imran Sheikh, Zubair Ahmed, Riyaz Sayyed, Andrea Mastinu

**Affiliations:** ^1^ Department of Plant Pathology, Dr. Khem Singh Gill Akal College of Agriculture Eternal University Baru Sahib India; ^2^ Department of Physics, Akal College of Basic Sciences Eternal University Baru Sahib India; ^3^ School of Physics and Materials Science Shoolini University Solan India; ^4^ Department of Botany and Microbiology, College of Science King Saud University Riyadh Saudi Arabia; ^5^ Department of Botany, Hindu College Mahatma Jyotiba Phule Rohilkhand University Bareilly India; ^6^ Department of Biological Science and Chemistry, College of Arts and Science University of Nizwa Nizwa Sultanate of Oman; ^7^ Department of Molecular and Translational Medicine, Division of Pharmacology University of Brescia Brescia Italy

**Keywords:** biocontrol, *Fusarium* oxysporum, nanocomposite, trichoderma

## Abstract

Soil‐borne plant pathogens are the most damaging pathogens responsible for severe crop damage. A conventional chemotherapy approach to these pathogens has numerous environmental issues, while biological control agents (BCAs) are less promising under field conditions. There is an immediate need to develop an integrated strategy for utilizing nanoparticles and biocontrol to manage soil‐borne pathogens, such as *Fusarium* wilt, effectively. Simulation of BCA metabolites to nanoparticle biocontrol metabolites is considered the most effective biocontrol approach. Combining Fe_2_O_3_ nanoparticles and *Trichoderma* in nursery and field conditions manages pathogens and increases plant growth characteristics. The present study evaluated the commercial biocontrol strains and the use of NPFe in combination with *Trichoderma harzianum* to enhance the biocontrol potential of *T. harzianum* against soil‐borne pathogens. The effectiveness of (NPFe + *T. harzianum*) was evaluated under in vitro conditions where combination was found most effective upto (87.63%) mycelial growth inhibition of pathogen and under field conditions lowest pooled *Fusarium* wilt incidence (19.54%) was recorded. Nanocomposites are beneficial for agricultural sustainability and environmental safety by upregulating the expression of genes linked to these processes, Fe NPs can activate plant defense mechanisms and increase plant resistance to pathogenic invasions. Additionally, as iron is a necessary component for plant growth and development, Fe NPs promote better nutrient uptake.

**Abbreviations**
NP: nanoparticle
NPFe: iron nanoparticle
PDA: potato dextrose agar

## 1 Introduction

Tomatoes are a commercially important crop grown in many countries, with an annual production of 177 million tons [1, 2]. In India, tomato production is about 20,708,000 metric tons annually [1]. The *Fusarium oxysporum* (F.o.) species can infect more than 100 host species, resulting in considerable losses in different agriculture and horticulture crops [3]. Pathogenic strains of F.o., both pathogenic and non‐pathogenic forms, are the cause of *Fusarium* wilt, a vascular wilt disease. *Fusarium* affects many key crops, such as tomato, flax, cucumber, melons, lettuce, strawberries, cotton, tobacco, carnation, and bananas, causing production losses due to many pathogenic strains of fungus [4]. One of the fungal diseases that threatens tomato plants globally and causes large financial losses in tomato production is wilt disease. This specific fungus can remain in the soil for 8–10 years in the form of resting structures called chlamydospores [5]. Crop yields are significantly impacted by *Fusarium* which can result in output losses of between 30% and 40%. In certain situations, these losses could potentially reach 80% if favourable weather patterns encourage the spread of fungi [6]. The frequency of *Fusarium* wilt disease in tomatoes was found to be as high in all districts of Uttar Pradesh, India where overall disease incidence varied between 10.67% and 80.34%. Similar findings were reported by Manikandan and Raguchander [7], who noted that in practically all tomato‐growing regions of Tamil Nadu State, India, the incidence of wilt disease ranged from 19% to 45%.

In India, every crop grown is susceptible to one or more species of wilt pathogen. Among various abiotic factors, iron (Fe) deficiency is the important nutrient for plant growth among the different other nutrients [8]. Fe is crucial to several vital physiological activities in plants, such as chlorophyll production, respiration, and other important physiological processes [9, 10, 11]. Other fields, such as biomedicine, water treatment, and post‐harvest losses used in magnetic materials, used Fe_2_O_3_ nanoparticles (NPs) [12]. Iron deficiency can severely damage plant growth and development [13, 14].

Metallic biogenic NPs have a lot of potential in agriculture, especially for pest control and fertilization [15]. Fungi such as *Aspergillus versicolor* and *Trichoderma harzianum* are used in several investigations for the production of silver NPs and utilization of these nanoparticles in the management of various pests and diseases [16, 17]. Biogenic NPs have many advantages, including the ability of fungi to cultivate on a large scale quickly and the high production of various important proteins and enzymes [16, 18]. *T. harzianum* is a fungus with a wide range of uses. *Trichoderma* can be used as a biological agent and, most importantly, utilized in the management of different phytopathogens, especially against various pathogens (*Fusarium*, *Pythium*, and *Phytophthora*) [19, 20, 21]. In the manufacturing of various biogenic NPs, *Trichoderma*, when utilized as a promoter agent, demonstrated promising outcomes in agriculture [22, 23]. According to the research, biogenic iron oxide NPs could help with biological pest control, leading to more sustainable farming practices [24, 25]. To achieve long‐term agricultural output with less environmental impact, several experts observed that NPs could be a good alternative for managing pests and diseases [26].

## 2 Materials and Methods

### 2.1 Collection and Isolation

For isolation, infected plant materials (10 days old plant) after transplanting of seedlings when true leaves about to start to form tomato were collected from the field, placed in plastic bags, and kept in the refrigerator at 4°C until used. The infected symptoms showed plant portions were placed on Petri dishes containing potato dextrose agar (PDA) after being gently cleaned with distilled water and surface sterilized for 1 min using a 1% sodium hypochlorite solution. *Fusarium* cultures kept pure on PDA plates were sub‐cultured for further use. *T. harzianum*, a biocontrol agent, was obtained from the Department of Plant Pathology, Eternal University.

### 2.2 Synthesis of Iron Nanoparticle (NPFe) With the Fungus *T. harzianum*

A culture of biocontrol *T. harzianum* culture was made using PDA media, and after that, 2 mL of *T. harzianum* suspension at (3 × 10^8^ cfu/g) concentration was prepared. After a 7‐day incubation period under aseptic circumstances at a temperature of 28°C, two bits of mycelium of 8 mm discs were introduced to 150 mL of potato dextrose broth [13]. For 16 days, the culture was shaken at 250 rpm at 25°C with an orbital shaker. The Whatman filter paper was used for the filtration of the biomass. The filtered material of *T. harzianum*, which contains various metabolites, enzymes, and reproductive structures to synthesize NPFe NPs, was used. The dried substance was discarded, and the aqueous filtrate was filtered to a final concentration of 1 × 10^−3^ mol L^−1^, forming the biocontrol NPFe using *T. harzianum*.

### 2.3 Characterization of NPFe

To determine structure and phase, the final products were subjected to tests at transmission electron microscopy (TEM), X‐ray diffraction (XRD), energy dispersive X‐ray spectroscopy (EDS) (Thermo Noran system SIX), Fourier transform infrared spectrophotometer (FTIR), field emission scanning electron microscopy (FE‐SEM).

#### 2.3.1 In Vitro Evaluation of (NPFe + *T. harzianum*), *Trichoderma hamatum,* and *T. harzianum*

The combined efficacy of (NPFe + *T. harzianum*) and the individual effect of *T. hamatum* and *T. harzianum* were evaluated under aseptic conditions [23] using PDA. Different concentrations of NPFe were separately sterilized. Before pouring equal amounts of double‐strength PDA media into Petri plates in an aseptic manner, NPFe (0.10 mg/mL) was added separately. After solidification, a 6 mm bit of *T. hamatum* and *T. harzianum* plates were inoculated in 7‐day‐old pure culture. After three replications of each treatment, inoculated plates were incubated at 28°C in a BOD. In addition, the biological activity of (NPFe + *T. harzianum*), *T. hamatum*, and *T. harzianum* against the pathogen *Fusarium* as a proof of concept was evaluated by the method given by [17, 27]. NPFe (0.10 mg/mL) was introduced separately (NPFe + *T. harzianum*) to equal volumes of double‐strength PDA medium before pouring into petri plates in an aseptic manner. *T. harzianum* and F.o. (test fungi) pathogen culture discs (6 mm diameter) were placed aseptically on the opposite side of each plate. Culture discs were collected from the edges of their growing pure culture colonies. Three times per treatment was applied, and then the cells were cultured in a BOD incubator at 25°C. After the test fungus reached its full size on control plates, the colony diameter of both test fungi was measured from two sides at a right angle to one another and compared to the control.

#### 2.3.2 Calculation of NPFe Minimal Fungicidal Concentration (MFC)

Observing the F.o. growth curve culture was grown in NB + 4% glucose broth medium, different NPFe doses were employed for antifungal tests. Every test was carried out in complete darkness. In this experiment, growth curves were produced by feeding a culture of F.o. (5 mL) with different doses of NPFe. At 600 nm, this culture's initial optical density (OD) was 0.1. Cultures were stirred (200 rpm) at 36.5°C during cultivation. The samples OD_600_ was monitored over time for calculating the growth curve. Further, the cells were centrifuged and resuspended in the fresh medium until they reached an OD_600_ of 0.1. The cells were cultured in NB + 4% glucose until they reached an OD_6__00_ (log phase) of 0.4–0.5 in the growth curve. The growth curve was developed by tracking the changes in the samples OD_600_ over time.

### 2.4 Histidine Inhibits the (F.o.) Antifungal Effect Caused by NPFe

The experiment aims to investigate the role of histidine and how it affects the growth curve of F.o. when NPFe is present in culture. Cultures were agitatedly and cultivated at 36.5°C (200 rpm). F.o. was a control grown on NB + 4% glucose broth medium. Cultures were agitatedly cultivated at 36.5°C (200 rpm). The growth curve was observed by tracking the sample's OD_600_ over time. F.o. was a control grown on NB + 4% glucose broth medium. At 36.7°C, cultures were agitatedly grown (∼200 rpm). The samples OD_600_ was monitored over time to determine the growth curve. F.o. was a control grown on NB + 4% glucose broth medium. The inhibitory effect of histidine on NPFe toxicity was examined following the incorporation of histidine into F.o. cultures containing NPFe (0.05 mg/mL). The role of histidine (1, 2, or 3 mM) on Fe NP toxicity was examined in F.o. cultures containing NPFe at 400 and 50 ppm dosages.

### 2.5 In Vivo Efficacy of (NPFe + *T. harzianum*) and Other Biocontrol Agents

Experiments for evaluating the combined efficacy of (NPFe + *T. harzianum*) and other important biological control agents were conducted at the Eternal University, Baru Sahib, in 2020 and 2021. Fungal antagonist *T. hamatum* was procured from the Department of Plant Pathology, Dr. Yashwant Singh Parmar, University of Horticulture and Forestry, India. First, a 25 g culture of pathogen F.o. produced in corn: sand meal (3:1) medium was incorporated into soil 10 days before the planting of tomato cuttings. *T. harzianum* mass culture was prepared by combining 300 mL of fermenter broth with 600 g of autoclaved talc powder and fermenting the mixture for 7 days in the molasses‐soy medium. The treatments included treating the soil with a talc‐based *T. harzianum* formulation by combining 20 g with 1 kg of farmyard manure (FYM). After planting, 1 kg of this combination was applied in seed rows of tomato nurseries, and NPFe was prepared in double distilled water (0.05% v/v) 550 mg^−^^1^ and applied as a soil drench [28]. Disease incidence was determined after every 10, 20, 30, and 40 days, and data was recorded on a percent basis:

$$\text{Disease incidence}\;(\%)=\frac{\text{Number of tomato plants infected}}{\text{The total number of tomato plants observed}}\times 100.$$

## 3 Results

### 3.1 Collection and Isolation of Pathogen

**Figure 1:**
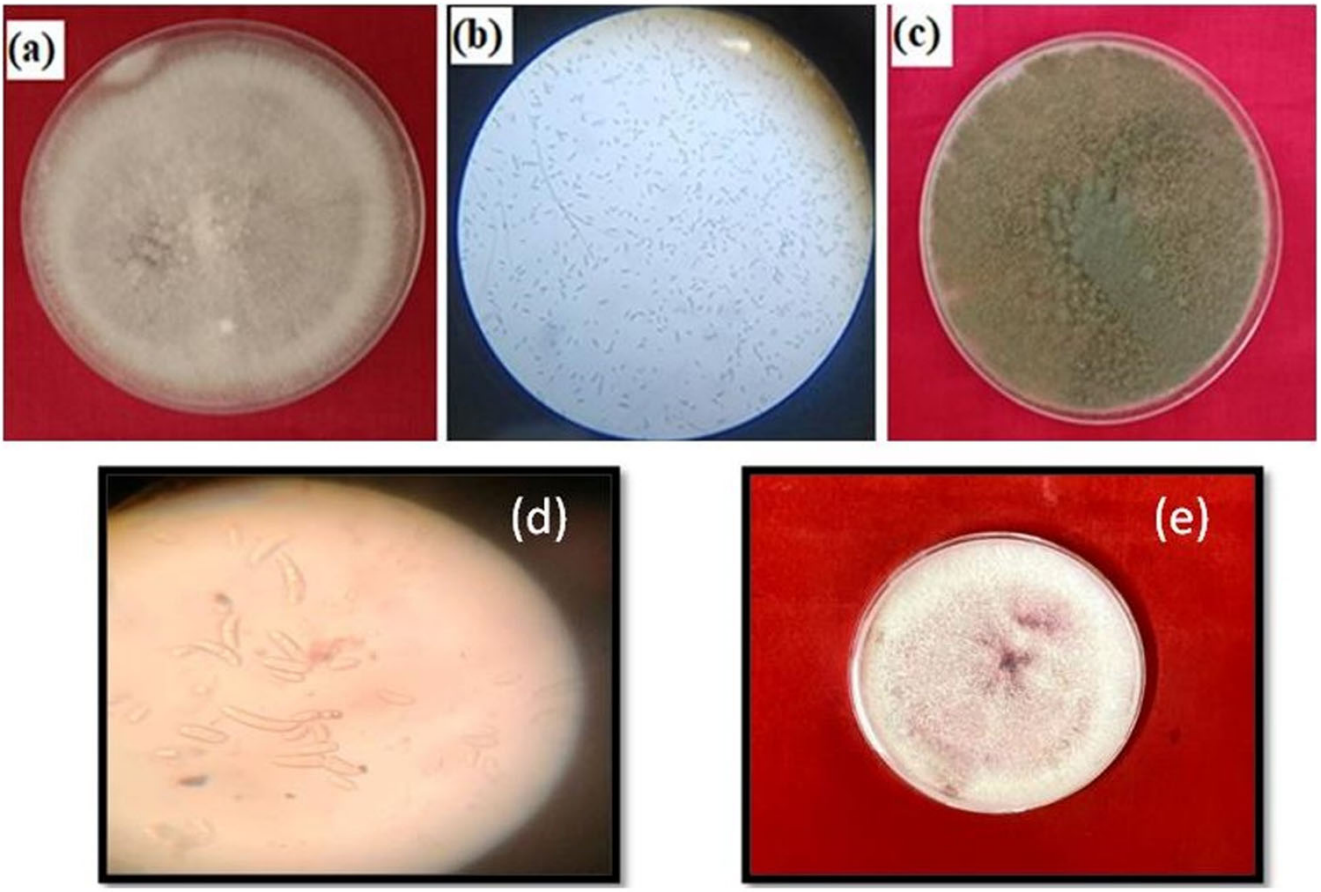
(a) Pure culture of *Fusarium oxysporum*; (b) asexual spores micro and macro conidia of *F. oxysporum*; (c) pure culture of *Trichoderma harzianum*; (d) macroconidia; (e) pure culture of *F. oxysporum*.

F.o. was isolated from the infected 10 days old tomato plant. The plant sample was inoculated on the sterile PDA and incubated at 28°C for 48 h [29]. Following the incubation, the resulting fungal growth was identified as F.o*.* based on color, texture, mycelium, micro and macroconidia, and other colony features (Figure 1).

### 3.2 Characterization

#### 3.2.1 XRD Analysis

**Figure 2:**
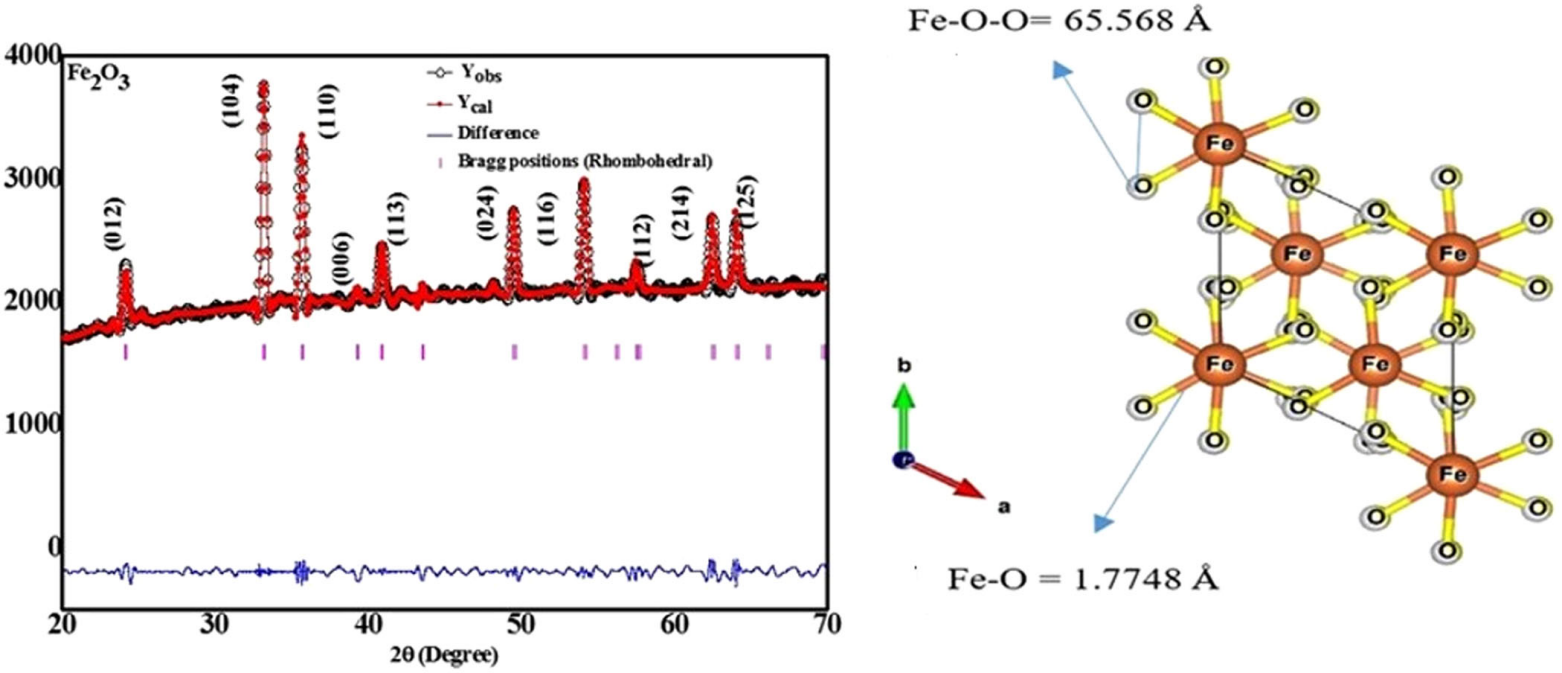
X‐ray diffraction of NPFe biogenically synthesized with the fungus *Trichoderma harzianum*.

Figure 2 represents the XRD analysis of biogenically synthesized Fe_2_O_3_ NPs. Peaks at 2θ values near 0.98°, 32.94°, 36.04°, 39.28°, 41.08°, 49.60°, 54.91°, 62.59°, 64.98°, and 66.06° belonged to crystallography planes demonstrate that Fe_2_O_3_ NPs were successfully designed and matched with the JCPDS card no. 882359. The atomic positions and lattice parameters were improved using Rietveld refinement on the XRD data, and the outcomes are displayed in (Supporting Information S1: Table S1).

There is good agreement between the estimated and observed patterns. The crystallite size obtained a value of ∼42 ± 7 nm using the Debye–Scherer equations below (Figure 2):

$$D=\frac{0.93\lambda }{{B\mathrm{cos\theta}}_{B}},$$

$$B={\left(B_{m}^{2}-B_{s}^{2}\right)}^{\frac{1}{2}}.$$

#### 3.2.2 FE‐SEM/TEM/EDS/FTIR Analysis

**Figure 3:**
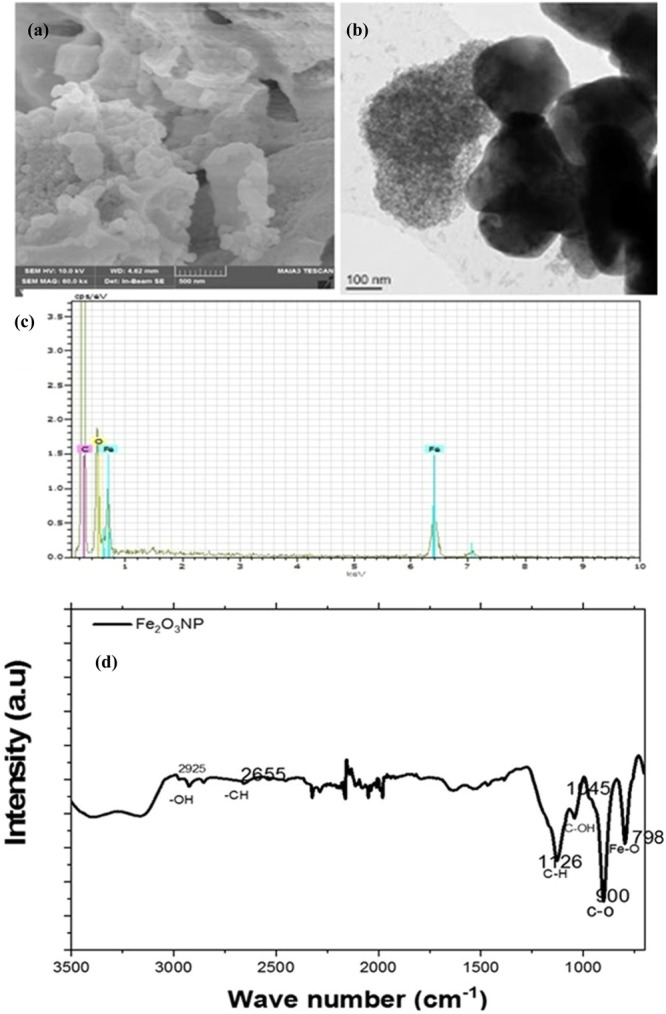
(a) SEM; (b) TEM; and (c) EDX study of the NPFe biogenically fabricated using fungus *Trichoderma harzianum*; (d) FTIR spectra obtained for the NPFe biogenically fabricated using the fungus *T. harzianum*.

The FE‐SEM micrograph (Figure 3a) shows flaks‐like particles and nucleated spherical NPs on larger grains. *T. harzianum* plays a role in green NP production, forming mesocrystals. These superstructures are crystalline NPs with rough outer surfaces and sizes ranging from nanometers to hundreds of micrometers. Biosynthesis processes prompt non‐classical nucleation events. TEM micrographs (Figure 3b) verified the manometer's particle size and demonstrated the NPs' transparency.

EDS (Figure 3c) depicted the stoichiometric presence of the predicted elements in the sample. Figure 3d depicts the absorption band of NPFe is more pronounced, with a sharp peak at 900 cm^−1^. The formation of NPFe is confirmed by Fe‐O starching at 797 cm^−1^, indicating extensive interactions between the filtrate and Fe_2_O_3_. Organic groups were found on the surfaces of NPs, possibly as a capping on the Fe‐O matrix [30].

### 3.3 In Vitro Evaluation of (NPFe + *T. harzianum*) and *T. harzianum* (Alone)

**Figure 4:**
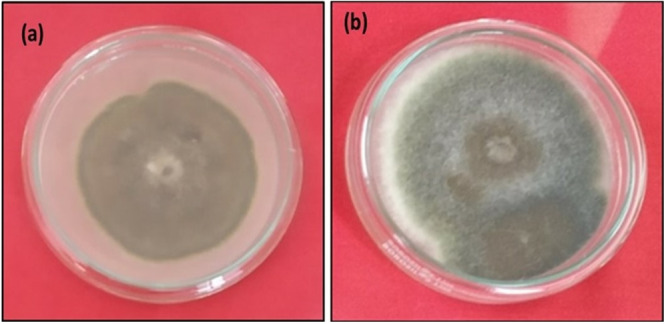
In vitro efficacy of (a) NPFe + *Trichoderma harzianum* (b) *T. harzianum* (alone).

The combined efficacy of (NPFe + *T. harzianum*) and *T. harzianum* alone were evaluated to check the growth of both treatments in the PDA medium (Figure 4). After a week of the incubation period, there is a presence of large number of growth in (NPFe + *T. harzianum*) and linear growth in *T. harzianum* alone. In this experiment, the growth of *T. harzianum* in the absence of NPs and the development of the *T. harzianum* along with the synthesized biogenic NPs (3 × 10^4^ conidia mL^−1^) were compared (Figure 4) [28, 29, 30].

### 3.4 In Vitro Efficacy of (NPFe + *T. harzianum*), *T. harzianum* (Alone), and *T. hamatum* Against *F. oxysporum* Wilt Pathogen of Tomato

**Figure 5:**
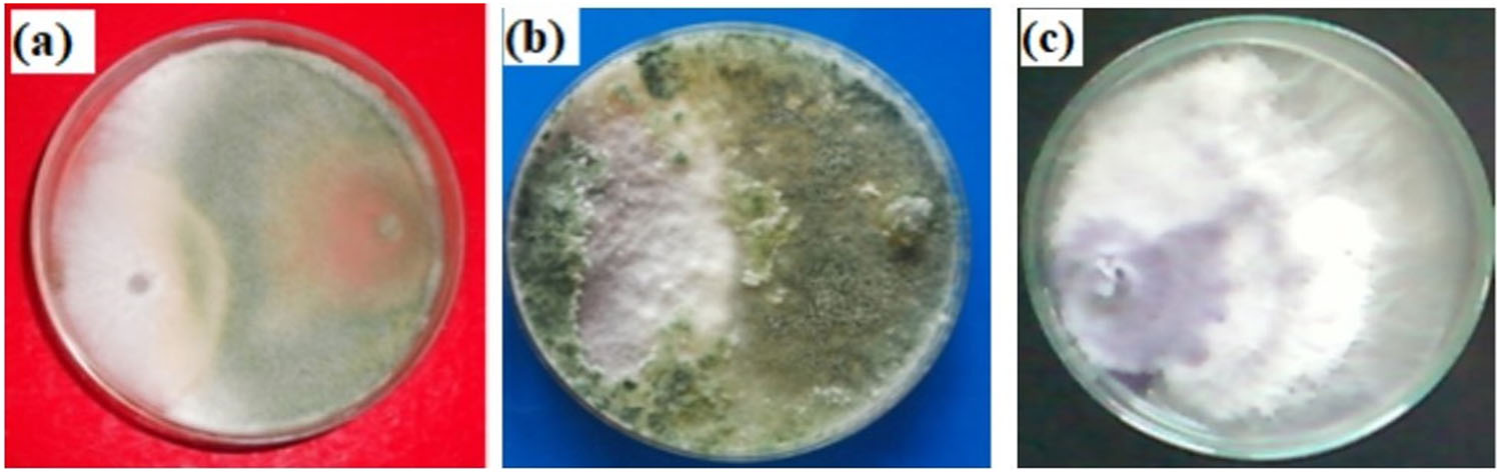
Antagonistic activity of (a) *Trichoderma harzianum*; (b) (NPFe + *T. harzianum*); and (c) control.

Antagonistic activities of (NPFe + *T. harzianum*), *T. hamatum*, and *T. harzianum* (alone) were evaluated against the *Fusarium* wilt pathogen (F.o.) (Table 1). Treatments when assessed under in vitro conditions, reduced the growth of the fungus ranging from 89.63% in (NPFe + *T. harzianum*), (87.63%) in *T. harzianum* and (52.77%) *T. hamatum* in comparison to control (Figure 5). The expression of chitinase genes chi42 and nag1 has been demonstrated to decrease disease in *Trichoderma* [31, 32, 33]. In a previously reported study, 12 isolates of *Trichoderma* were evaluated and produced more lytic and nonlytic enzymes when being exposed to *Aspergillus niger* and the antagonist, showing that these enzymes are involved in the battle against phytopathogens [20]. A study on the use of bio‐fabricated Fe_2_O_3_ NPs for controlling citrus brown rot using scanning electron microscopy [34]. The best mycelial inhibition was noted at 1.0 mg/mL concentration of Fe_2_O_3_ NPs.

**Table 1:** In vitro efficacy of (Fe NPs + *Trichoderma harzianum*), *Trichoderma hamatum,* and *T. harzianum* (alone) against *Fusarium oxysporum* wilt pathogen.

Pathogen	Mycelial growth inhibition (%)			
	*T. hamatum*	*T. harzianum*	FeNPs + *T. harzianum*	Control
*F. oxysporum*	52.77 ± 0.58 (7.33 ± 0.04)	87.63 ± 0.60 (9.41 ± 0.03)	89.63 ± 0.59 (9.52 ± 0.03)	91.99 ± 0.10 (10.04 ± 0.05)

C.D.	0.105
SE_(m)_	0.033
SE_(d)_	0.046
C.V.	0.641

*Note:* Value are the mean of triplicates. ± = Standard deviations.

### 3.5 Calculation of NPFe MFC

**Figure 6:**
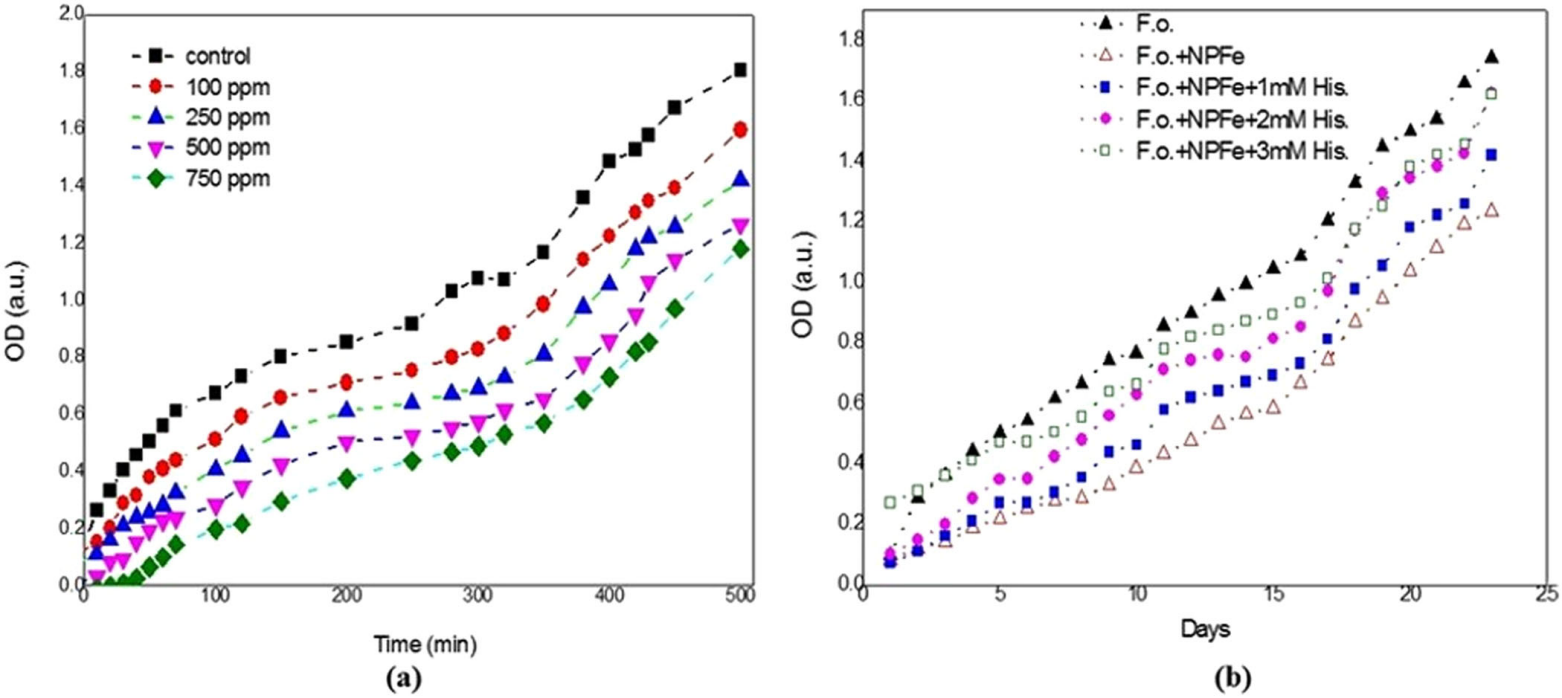
(a) Determination of minimal fungicidal concentration (MFC) of NPFe; (b) the antifungal activity of NPFe on *Fusarium oxysporum* is inhibited by histidine.

The present study indicates that NPFe NPs suppressed F.o. growth in a concentration‐dependent manner. F.o. was cultured in suspension without adding NPFe as the negative control. As the concentration of NPFe rise, Figure 6a lesser number of fungus cells were present. The results matched the OD of the developing cultures as the number of viable cells from growing cultures at various growth stages is calculated and counted by plating method. NPFe inhibited the development of F.o*.* when added during the logarithmic phase.

### 3.6 The Antifungal Effect of Npfe on (F.o.) Is Inhibited by Histidine

The antifungal effect of NPFe on (F.o*.*) is inhibited by histidine. The Role of ROS in NPFe due to a reduction in the mycelial growth inhibition of the F.o. pathogen was examined in this experiment. The F.o. culture was used in an experiment to test the effects of histidine at concentrations of 1, 2, and 3 mM on NPFe‐mediated toxicity. Figure 6b demonstrates how histidine affects the F.o. growth curve when NPFe (0.05 mg/mL) is present. When NPFe is present in the culture, histidine has a dose‐dependent impact on the F.o. growth curve as observed in (Figure 6b).

### 3.7 In Vivo Efficacy of (NPFe + *T. harzianum*), *T. harzianum* (Alone), and *T. hamatum* Against *F. Oxysporum* Wilt Pathogen

**Figure 7:**
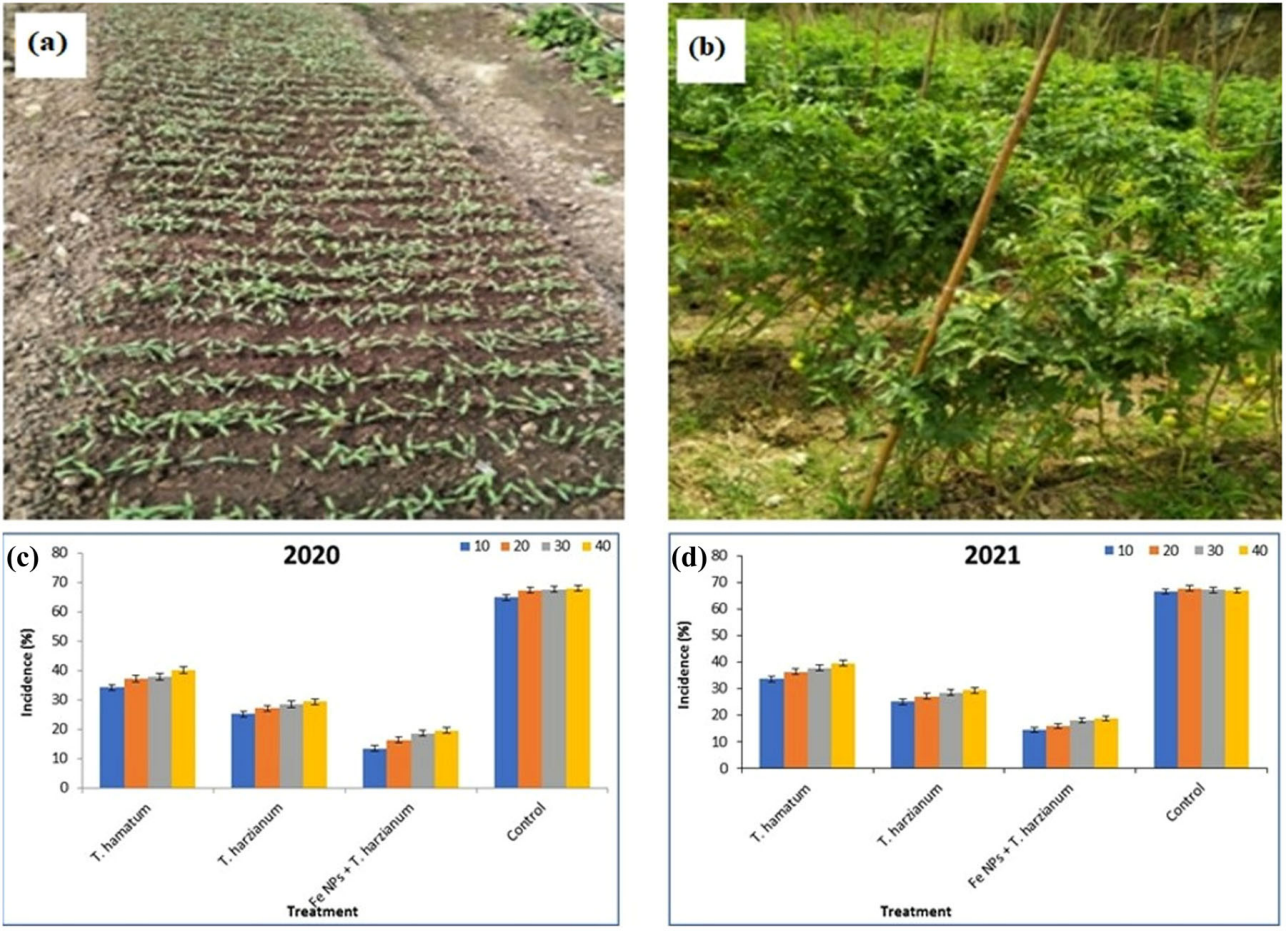
Field evaluation of NPFe + *Trichoderma harzianum* and other biocontrol agents against *Fusarium* wilt pathogen. (a) Day 10; (b) Day 40; (c) in 2020; (d) in 2021.

The study evaluated the effectiveness of NPFe + *T. harzianum* all other biocontrol agents in the vegetable farm of Eternal University between 2020 and 2021. The results showed NPFe + *T. harzianum* was the most effective in inhibiting the mycelial growth of wilt pathogen by 16.92% and *T. harzianum* by 27.49% (Figure 7). After 40 days of planting, the NPFe + *T. harzianum* (19.54%) treatments had the lowest pooled *Fusarium* wilt incidence and in *T. harzianum*, the incidence of wilt disease was 29.31%. *Fusarium* wilt disease incidence was highest (67.89%) in the control treatment after 40 days of planting (Table 2).

**Table 2:** Field evaluation of *Trichoderma hamatum*, *Trichoderma harzianum,* and NPFe + *T. harzianum* against wilt pathogen (*Fusarium oxysporum*).

Treatment	Incidence (%) of wilt pathogen at different intervals (days)									
	2020					2021				
	10	20	30	40	Mean	10	20	30	40	Mean
*T. hamatum*	34.25 (35.80)	37.19 (37.53)	37.81 (37.92)	40.12 (39.28)	37.34 (37.64)	33.65 (35.44)	36.39 (37.08)	37.81 (37.92)	39.52 (38.93)	36.84 (37.34)
*T. harzianum*	25.11 (30.05)	27.04 (31.32)	28.50 (32.25)	29.31 (32.76)	27.49 (31.59)	25.11 (30.05)	27.04 (31.32)	28.50 (32.25)	29.31 (32.76)	27.49 (31.60)
NPFe + *T. harzianum*	13.34 (21.40)	16.27 (23.77)	18.55 (25.49)	19.54 (26.22)	16.92 (24.22)	14.44 (22.08)	15.88 (23.47)	18.06 (25.13)	18.79 (25.67)	16.72 (24.09)
Control	64.86 (53.64)	67.33 (55.37)	67.56 (55.25)	67.89 (55.87)	66.15 (54.42)	66.57 (54.67)	67.73 (55.37)	67.13 (55.01)	66.85 (54.84)	66.85 (54.84)
Mean	34.39 (35.22)	37.06 (37.00)	38.10 (37.73)	38.78 (38.17)		34.87 (35.56)	36.76 (36.81)	37.58 (37.40)	38.64 (38.09)	
Control: water spray (800 L/ha)										
C.D._0.05_										
(2020)										
			C.D.			SE_(d)_			SE_(m)_	
Days			0.69			0.34			0.24	
Treatment			0.69			0.34			0.24	
Days × treatment			1.38			0.69			0.48	
(2021)										
Days			0.63			0.31			0.22	
Treatment			0.63			0.31			0.22	
Days × treatment			1.26			0.63			0.44	

*Note:* Values are the mean of triplicates. The Figure in parentheses are angular transformed values.

## 4 Discussion

NPFe did not hinder the growth of *Trichoderma* sp. at (550 ppm) concentrations; on the contrary, low concentrations of NPFe promoted tomato plant growth and yield. An experiment was conducted to evaluate the in‐vitro efficacy of various biocontrol agents against *Alternaria solani*, the pathogen that causes early tomato blight. The addition of NPs improved plant growth characteristics, production, and yield in potatoes, soybeans, cabbage, and cauliflower at deficient concentrations without causing any phytotoxic effects in plants [32].

Recent research indicates that applying Fe_2_O_3_ NP as a nanofertilizer to peanuts (*Arachis hypogaea*) increases the amount of phytohormone content, biomass, root and stem length, antioxidant enzymes, and plant height [33]. Future advancements in crop production and disease management could be achieved by utilizing Fe_2_O_3_ NPs for enhanced quality yields [35]. The magnetic properties of FeNPs as nanofertilizers are due to their nanosize and the process reduced organic materials to inorganic complex molecular compounds [1]. Similar results were observed in SiO_2_‐NPs exhibited promising antifungal activity against *A. solani* by minimizing disease incidence from 82.5% to 25% over control at 100 ppm [36].

AgNPs were evaluated against *Alternaria porri* at various concentration at 150 ppm (91.71%), and the highest percentage of inhibition was observed [37]. Green‐synthesized silver nanomaterials (AgNMs) from wild gourd (*Citrullus colocynthis*) and rough cocklebur (*Xanthium strumarium*) and applied them at three different concentrations to evaluate their efficacy against early tomato blight [38]. The findings showed that AgNMs based on *C. colocynthis* were the most successful, lowering the disease incidence of *A. solani* to 22%. Additionally, these AgNMs increased the quantity of fruits per plant and greatly improved tomato yield by 13%. Numerous factors can influence the stability and toxicity of NPs in biological systems. The size of NPs is a significant factor among these. In other words, their size frequently affects their physico‐chemical characteristics. Because of their greater surface area, smaller NPs have shown more toxicity to plants than bigger NPs [39]. But when it comes to normal HEK293 cell lines, Fe_2_O_3_ exhibits dose‐dependent (400 μg ml^−1^) cytocompatibility. They cannot cause any ROS activity inside cells up to this dosage) [40].

Nanotechnology has a number of uses in agriculture that could transform traditional farming practices and solve many agriculture‐related problems [41, 42, 43]. By using nanoparticles to deliver active substances precisely, less pesticides and fungicides are needed, the release period is prolonged, and off‐target effects are reduced, all of which help to lessen environmental damage [44, 45, 46, 47]. FeNPs nowadays are utilized due to their high biodegradability, easily decompose into smaller, non‐toxic compounds over time. This characteristic is advantageous for environmental safety and the sustainability of agricultural practices. Notably, Fe NPs can activate plant defense mechanisms by upregulating the expression of genes associated with these processes, thereby enhancing plant resistance to pathogenic invasions. This dual functionality underscores the potential of Fe NPs in promoting both plant health and sustainable agriculture.

The use of nanoparticles as nanofertilizers or nanopesticides has both shown beneficial and negative effects on plant‐associated microbial populations as well as crop properties as reported by many researchers if they are used in proper concentrations and dozes [48]. The combined use of Fe_2_O_3_ and *Trichoderma* in nursery and field conditions increases plant growth characteristics while also assisting in managing soil‐borne diseases. Chemicals and various fertilizers used in agriculture are hazardous to human health, so the effectiveness of NPFe + *T. harzianum* as a nanofertilizer can replace chemical fertilizers in the future [45, 49].

## Author Contributions

**Sushma Sharma:** formal analysis, methodology, writing–original draft. **Poonam Kumari:** project administration, supervision, writing–review and editing. **Mamta Shandilya:** formal analysis, writing–review and editing. **Sapna Thakur:** formal analysis, methodology, writing–original draft. **Kahkashan Perveen:** formal analysis, funding acquisition, writing–review and editing. **Imran Sheikh:** investigation, writing–original draft, writing–review and editing. **Zubair Ahmed:** formal analysis, writing–review and editing. **Riyaz Sayyed:** formal analysis, writing–original draft, writing–review and editing. **Andrea Mastinu:** funding acquisition, data curation, formal analysis and writing–review and editing.

## Conflicts of Interest

The authors declare no conflicts of interest.

## Supporting information

Supporting information.

## Data Availability

All the data of the study are included in the manuscript and its Supporting Information.
