# Supplementary material for: The Combination of α‐Fe2O3 NP and Trichoderma sp. Improves Antifungal Activity Against Fusarium Wilt
Source: J Basic Microbiol. 2025 Jan 19;65(4):e2400613. doi: 10.1002/jobm.202400613 (PMC11973845; doi:10.1002/jobm.202400613)
Supplement: Supplementary file 1 — Supporting information. [file JOBM-65-e2400613-s001.docx]

**Table S1** The refined cell parameters, cell volume, R factors, and goodness of fit parameter (χ²) for Fe₂O₃ nanopowder

| **Simulated parameters** | | | **Fe_2_O_3_** |
| --- | --- | --- | --- |
| Rhombohedral, lattice parameter | a = b (Å) |  | 5.0323 |
|  | c (Å) |  | 13.7478 |
| Rhombohedral, unit cell volume | V (Å) |  | 301.505 |
| Atomic Positions  Rhombohedral | Fe | x | 0.0000 |
|  |  | y | 0.0000 |
|  |  | z | 0.35530 |
|  | O | x | 0.69400 |
|  |  | y | 0.0000 |
|  |  | z | 0.25000 |
| R-factor (%) Rhombohedral  GoF | | R_p_ | 34.7 |
|  |  | R_exp_ | 53.7 |
|  |  | R_wp_ | 22.4 |
|  |  | R_Bragg_ | 2.06 |
|  |  | χ^2^ | 0.98 |
